# The Effect of Vitamin D Deficiency on Overgrowth of Uterine Fibroids: A Blinded Randomized Clinical Trial

**DOI:** 10.22074/IJFS.2020.134567

**Published:** 2021-03-11

**Authors:** Fatemeh Davari Tanha, Elham Feizabad, Maryam Vasheghani Farahani, Hoora Amuzegar, Behnaz Moradi, Saghar Samimi Sadeh

**Affiliations:** 1 Department of Obstetrics and Gynecology and Reproductive Endocrinology, Vali- asr Health Research Center, Vali- asr Hospital, Tehran University of Medical Sciences, Tehran, Iran; 2 Department of Obstetrics and Gynecology, Yas Hospital, Tehran University of Medical Sciences, Tehran, Iran; 3 Department of Community Medicine, School of Medicine, Tehran University of Medical Sciences, Tehran, Iran; 4 Department of Radiology, Yas Hospital, Tehran University of Medical Sciences, Tehran, Iran; 5 Department of Anesthesiology, Yas Hospital, Tehran University of Medical Sciences, Tehran, Iran

**Keywords:** Cell Proliferation, Dietary Supplements, Leiomyoma, Premenopausal Women, Vitamin D

## Abstract

**Background:**

To evaluate the effects of vitamin D (vitD) supplement on uterine fibroid growth.

**Materials and Methods:**

A randomized blinded clinical trial was conducted at a tertiary university-based hospital from August 2017 to September 2018. Totally, 204 women were enrolled into the study. They had at least one uterine fibroid >10 mm on transvaginal ultrasound and their vitD level was insufficient (i.e. 20-30 ng/ml). The intervention group was treated with vitD 50000 U supplements for two months. After 2 months, ultrasound screening and vitD level measurement was done in both groups.

**Results:**

At first, the mean serum vitD levels in intervention and control group were 23.62 and 23.20 ng/ml, respec- tively. After 8 weeks, the mean serum vitD levels in the control and intervention group were 22.72 and 28.56 ng/ml respectively (P<0.05). Also, mean fibroma diameter in the intervention group before and after 8 weeks of vitD supple- mentation was 43 ± 4.68 and 42.6 ± 1.31 mm, respectively. Mean uterine fibroid diameter in the control group which did not receive vitD supplements, before and after 8 weeks was 41.98 ± 5.25 and 47.81 ± 3.42 mm, respectively. The variation in the mean size of the uterine fibroid between the control and intervention group which was respectively about 5.83 mm increase and 0.48 mm decrease, was significant (P<0.001).

**Conclusion:**

Our results showed that vitD supplementation prevents fibroid growth. It seems that vitD supple- ment is a simple, safe and inexpensive modality for leiomyoma growth prevention (Registration number: IRCT201703122576N15).

## Introduction

In women of the reproductive age, uterine fibroids (leiomyoma) are monoclonal is a benign and most common gynecological tumor that depends on hormonal changes (1-5). These tumors affect 25-80% of reproductive age women depending on their demographic characteristic [race, age, and body mass index (BMI)], past medical history [hypertension (HTN), infertility, and premenopausal period], nutritional habits (use of food additive or soy bean milk) and family history (6-9).

It has been reported that some intrinsic abnormalities during the menstrual period including abnormal myometrial receptors for estrogen and hormonal changes or altered responses to ischemic injury, may be accountable for the start of (epi) genetic changes found in uterine myomas (10, 11).

Most uterine fibroids are asymptomatic, but they can cause symptoms, according to their size and location, like abnormal or heavy menstrual bleeding, iron deficiency anemia, bloating, constipation, urinary symptoms, pelvic pain, problems in intercourse, or pregnancy complications like recurrent miscarriage, premature labor and infertility (5, 12-16).

Symptomatic tumors are mainly treated by surgery. In addition to high direct costs of fibroma surgery treatments, these treatments are not suitable for women who wish to maintain fertility. However, nonsurgical treatments are approved just for short-term treatment and have their own disadvantages (5, 17). On the other hand, some therapeutic agents like gonadotropin hormone-releasing hormone (GnRH) analogue, oral GnRH antagonist (Elagolix), selective progesterone receptor modulators (SPRM), vitamin D (vitD) and green tea extracts are candidates to blunt the fibroid growth (18, 19).

Of medical therapeutic agents, vitD is a safe, inexpensive and available treatment without major side effects in therapeutic dose, which is assumed theoretically as an antitumor agent (20). It modulates gene (like cell growth and division genes and encoding estrogen and progesterone receptors genes) expression via vitD receptor and can result in regulation of cellular proliferation and cellular differentiation, stimulation of apoptosis that finally results in inhibition of malignant transformation and prevention of tumor cells proliferation (21, 22). Exposure to 1α, 1,25(OH)_2_ D_3_ inhibits the growth of melanoma, and lung, colon, prostate and breast cancer (23-27). So, vitD can be assumed as a preventive tool for high-risk group of patients that are susceptible to uterine fibroma (28).

Since, there is a high prevalence of vitD deficiency in Iran, there are interesting articles about the theoretical role of vitD in growth inhibition of uterine fibroid and the present data are inadequate to show the role of vitD as a medical therapy for the treatment of uterine fibroids, there is a need for a clinical trial that can assess inhibitory effects of vitD on the uterine fibroid size. Therefore, we conducted a randomized blinded clinical trial for evaluating the effects of vitD on the leiomyoma mean diameter in women with at least one uterine fibroid>10 mm referred to gynecology clinic in Tehran University of Medical Sciences’ Hospital.

## Materials and Methods

This randomized blinded clinical trial was conducted between August 2017 and September 2018 in a tertiary university-based hospital.

All reproductive age women who referred to gynecologic clinic and had at least one uterine fibroid>10 mm in transvaginal ultrasound, were evaluated using a blood sample for vitD levels. According to the Endocrine Society Practice Guideline on vitD status, deficiency was defined as vitD<20 ng/ml, insufficiency as 21-29 ng/ml, and sufficiency as at least 30 ng/ml (29).

All the patients with uterine fibroid and insufficient levels of vitD (21-29 ng/ml) were included in the present study. Due to ethical concerns, patients with deficient levels of vitD (<20 ng/ml) were excluded from the study and received vitD as treatment. Other exclusion criteria were refusing follow-up visit or being candidate for hysterectomy or myomectomy due to related symptoms of uterine fibroid like bleeding, pain, pregnancy, menopause, use of oral contraceptive during the last 3 months, consuming vitD supplements during the last 3 months, having BMI of <18 or >30 kg/m^2^ (30) and malignancy

**Fig.1:**
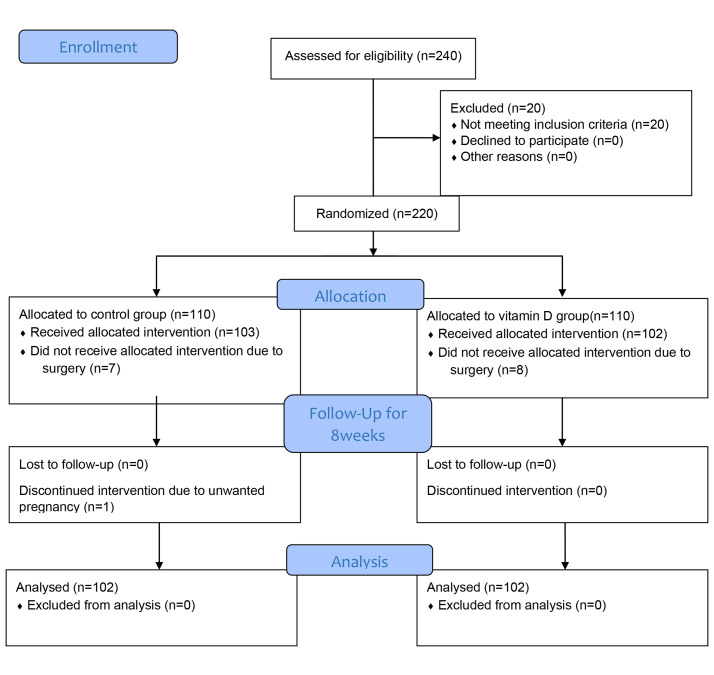
Consort flow diagram of study.

Two-hundred and forty women with uterine fibroid were referred to the clinic. Of them, 20 patients had sufficient levels of vitD, and seven patients underwent surgery in the control group and eight patients in the intervention group and one woman with unwanted pregnancy was excluded from the control group during the study. To tally 204 women, 102 in each group, completed the study protocol and data was analyzed (according to intention to treat analysis) (Fig.1).

The vitD level was measured by a Kit (Narvanteb Company Kit, Iran) based on a chemiluminescence technology. Each patient had two blood samples, one in the initiation of the study and one after 8 weeks of observation or treatment with vitD in the same laboratory following the same method.

According to the sample size formula (α=0.05, β=90%, r=-0.31), we needed 212 patients for this study (106 patients for each groups) (30). Randomization of the patients was done based on block randomization by a computer program (Random Allocation Software) that was performed by a secretory that was blinded to the study group.

The uterine fibroid size was measured by a radiologist who was experienced in gynecologic ultrasound in the initiation of study and after 8 weeks of treatment with vitD supplement for both study groups. The radiologist was blinded to the grouping. Uterine fibroid was defined as well-defined, hypoechoic, heterogenous mass. Regarding the anatomical site in the uterus, patients divided as subserosal if the development was in the outer portion of uterus, intramural if the leiomyoma localized in the myometrium, and submucosal if the development was in to uterine cavity. The leiomyoma was measured by three perpendicular diameter and the mean of them was calculated. The scans were performed by a 5.5-MHZ probe of Voluson 730 GE Healthcare, Milwaukee, WI.

The intervention group with insufficient levels of vitD, received vitD 50000 IU (D-Vigel 50000, DaanaPharma Company, Iran) orally once per a week for 8 weeks and then a blood sample for vitD levels measurement was collected and the second transvaginal ultrasound was performed (1 to 2 weeks after the last dose of vitD). The control group with insufficient levels of vitD was observed for 8 weeks without vitD supplementation and 1 to 2 weeks after that the second blood sample was collected and transvaginal ultrasound was performed, then they received vitD supplement to reach sufficient vitD concentration.

Finally, variations in the mean diameter of uterine fibroid and vitD levels were measured and compared between thetwogroup as the primary outcomes.

### Statistical analysis

All the statistical analyses were performed using SPSS version 24.0 (IBM, New York, USA). Quantitative data are expressed as mean ± standard deviation and categorical data are expressed as frequency and percentage. The Kolmogorov-Smirnov test was used to evaluate the distribution of the data. Independent samples, one-sample t test and Crosstabs were used in assessing the variable relationship. P<0.05 was considered significant.

### Ethical considerations

The study was approved by the local Ethical Committee (IR.TUMS.VCR.REC.1396.2701) and the trial was registered as IRCT201703122576N15. All the patients signed the informed consent before being enrolled in to the study groups.

## Results

Totally, 204 women, 102 in each group, completed the study protocol, and data were analyzed. The mean age and BMI in the control and intervention group were 37.21 and 34.89 years and 26.71 and 25.63 kg/m^2^ respectively. About 6.86% of women had a history of infertility in the intervention group versus 5.88% in the control group. The group were not statistically different in terms of age, BMI and history of infertility (Table 1). 

The mean vitD levels in the control and intervention group was 23.62 and 23.20 ng/ml, respectively which was not statistically different. At the end of the study, the mean vitD levels in the control and intervention group was 22.72 and 28.56 ng/ml, respectively.

**Fig.2:**
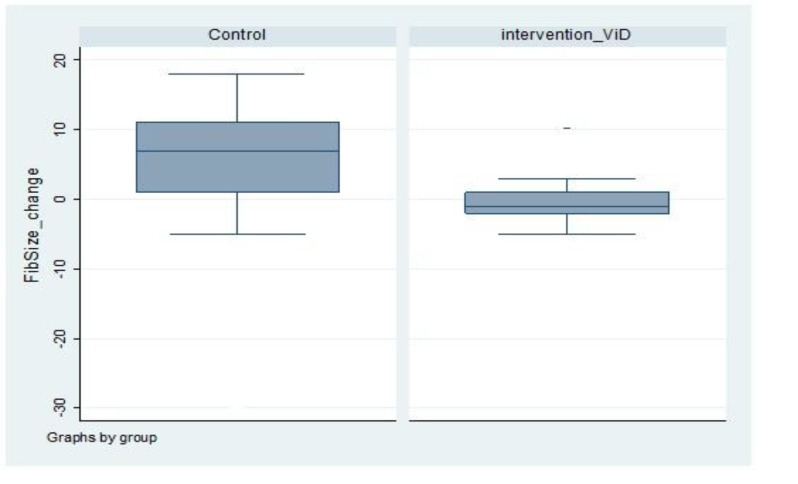
Diagram of variations in the mean diameter of uterine fibroid in the two groups.

The mean uterine fibroids diameter in the control group before and after the study was 41.98 ± 5.25 and 47.813 ± 3.42 mm, respectively. The mean uterine fibroid size increased for 14.5% in control group. Mean vitD levels before and after the study were 23.6 and 22.7 ng/ml, respectively in the control group. It showed 0.9 unit or 4% decrement; the levels were assumed stable, if the laboratory error was considered (Fig.2).

**Table 1:** Demographic data of women in the two groups


Variable	Control group	Interventiongroup	P value

Age (Y)	37.2 ± 91.13	34.89 ± 10.10	0.080
BMI (kg/m^2^)	26.7 ± 2.51	25.63 ± 3.31	0.090
History of infertility	6	7	0.770
Vitamin D level before (ng/ml)	23.62 ± 4.80	23.20 ± 4.64	0.520
Vitamin D level after (ng/ml)	22.72 ± 5.21	28.56 ± 5.04	0.001
Mean leiomyoma diameter before (mm)	41.98 ± 11.31	43.09 ± 14.36	0.180
Mean leiomyoma diameter after (mm)	47.81 ± 14.13	42.61 ± 15.53	0.018
Subserosal leiomyoma	44 (43.14)	50 (49.02)	0.561
Intramural leiomyoma	45 (44.12)	43 (42.16)	
Submucosal leiomyoma	13 (12.75)	9 (8.82)	


Data are presented as mean ± SD or n (%). BMI; Body mass index.

Regarding the site of uterine fibroids in the control group, 43.14% of them were subserosal, 44.12% intramural and 12.75% submucosal. In the intervention group, 49.02% were subserosal, 42.16% intramural, and 8.82% submucosal uterine fibroid.

The most common type of uterine fibroids in the control group was intramural followed by subserosal, versus the intervention group in which, the subserosal was the most common type followed by intramural. The submucosal leiomyoma had the lowest incidence in both groups.

**Fig.3:**
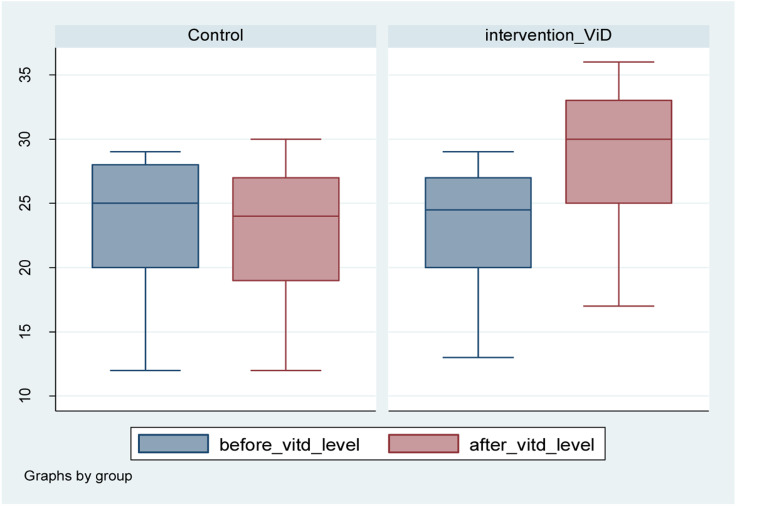
Diagram shows vitamin D levels in both group, before and after the study.

The mean uterine fibroid size in the intervention group before and after the study was 43.09 ± 14.36 and 42.61 ±15.53 mm, respectively. The size decreased to 0.48 mm. Mean vitD levels before and after intervention qwew23.2 and 28.5 ng/ml, respectively. It showed 5.36 unit or 24% increase in vitD levels (Fig.3).

The change in the size of the uterine fibroid between the intervention and control group had a significant difference, the mean changes in size was 5.83 mm in the control group compare to - 0.48 mm in the intervention group (P=0.001). The control group had increases in size versus a slight decrease in the intervention group (Table 2).

**Table 2:** Comparison of uterine fibroid size and vitD level changes in the two groups after the intervention


Groups	Uterine fibroid size (mm)	% Size change (ng/ml)	Vitamin D	% Size change

Control n=102	5.833 ± 0.642	14.5	-0.902 ± 1.29	-4
Intervention n=102	-0.480 ± 0.246	-2.5	5.362 ± 2.567	24
P value	<0.001		<0.001	


Data are presented as mean ± SD or %.

The mean uterine fibroid size changes in the control group regarding the type of uterine fibroid was 5.15,7.33, and 2.92 mm for subserosal, intramural, and submucosal, respectively. Maximum change in mean leiomyoma diameter was found for intramural followed by subserosal. 

The mean uterine fibroid size changes in the intervention group was 0.3 mm decrease for intramural and subserosal but for submucosal 2.33 mm decrease in size occurred after the intervention.

For evaluating the error of measurement of uterine fibroid diameter, another analysis was done and it showed that the mean change was 4.54 and -0.17 mm for the control and intervention group, respectively (P=0.001).

## Discussion

This trial showed that vitD given to at a therapeutic dose vitD insufficient women, can inhibit the leiomyoma growth which was detectable by ultrasound; however, women in the control group (who did not receive vitD treatment) had some growth in leiomyoma size.

There are some articles about the role of vitD as antitumoral agent (7, 9, 17, 19, 28). The receptor of vitD is a nuclear receptor which is activated by 1, 25(OH)_2_ D_3_ , resulting in modulation of the transcription rate of target gene (like cell growth and division genes and estrogen and progesterone receptors encoding genes) (31). These functions are the orgin of anti-tumor effects of 1,25(OH)_2_ D_3_ on uterine fibroid.

Accumulating evidence suggests that the metabolic pathways of vitD may play a key role in the developing of several gynecological diseases. Indeed, the vitD receptor (VDR)-mediated signaling pathways and vitD levels seem to (deeply) affect the risk of polycystic ovary syndrome (PCOS), endometriosis, infertility, ovarian and even breast cancer, and affect a woman’s response to menopausal status (32-35).

Also another study evaluated vitD concentration and uterine fibroid in premenopausal women by ultrasound (36). About 90% of black women and 50% of white women had insufficient vitD levels. These women had 62% chance of uterine fibroid in comparison with women who had sufficient vitD concentrations.The study was showed that sufficient levels of vitD are associated with decreased risk of liomyoma. VitD concentration was checked from stored plasma of patients and 620 blacks and 416 whites were evaluated. Their samples were randomly chosen from National Institue of Environmental Health Sciences Uterine Fibroid Study during 1996- 1999. Women were asked regarding sun exposure by a questionaire that evaluted sun exposure>1 hour/day

Another cross-sectional study (37) showed that 52 women with uterine fibroid diagnosed by magnetic resonance imaging (MRI) or ultrasound had vitD level<30 ng/ml. The study showed that 85% of women with documented uterine fibroid were vitD deficient and that confirmed our study results.

Another prospective cross-sectional study in Turkish premenopausal women showed that traditional costume, being a house wife and low eduction are risk factor for vitD deficiency. Also, the study showed that vitD defficiency in women with leiomyoma was more prevalent versus in women without leiomyoma (38).

Moreover, a study by Mitro and Zota (39) in 3590 women with uterine leiomyomata, in the National Health and Nutrition Examination Survey indicated that there was no relationship between vitD and odds of uterine fibroids. Although, subgroup analysis performed on the same data showed that insufficient serum vitD was associated with significantly higher odds of uterine fibroids in white women but not in black patients.

*In vivo* vitD production is affected by sun exposure, geographical locations, latitude, season, weather condition, clothing and use of sunscreens have an important effect on vitD level. Indoor activity and dark skin are risk factor for black house-wife women for having fibroma (28, 40).

The strengths of our study were the design as a randomized clinical trial and accurate inclusion and exclusion criteria. The limitation of the present study is the short follow-up period (i.e.8 weeks). We recommend a clinical trial with longer period of follow-up (i.e. 12 months) for further measurement of leiomyoma size. Another limitation was that due to ethical considerations, we could not include women with vitD<20 ng/ml in the study and compare the effect of treatment on these women with the control group, and the reluctance of patients to participate in the study were another limitations of this study

## Conclusion

Our results showed that vitD supplementation prevents fibroid growth. It seems that vitD supplement is a simple, safe and inexpensive modality for leiomyoma growth prevention.
